# Incremental value of PET and MRI in the evaluation of cardiovascular abnormalities

**DOI:** 10.1007/s13244-016-0494-5

**Published:** 2016-05-25

**Authors:** Hamid Chalian, James K. O’Donnell, Michael Bolen, Prabhakar Rajiah

**Affiliations:** Department of Radiology, University Hospitals Case Medical Center, Cleveland, Ohio USA; Cardiovascular Imaging Laboratory, Imaging Institute, Cleveland Clinic Foundation, Cleveland, Ohio USA; Cardiothoracic Imaging, Department of Radiology, UT Southwestern Medical Center, 5323 Harry Hines Boulevard, Dallas, Texas 75390 USA

**Keywords:** PET, MRI, Cardiac, Ischemia, Neoplasm

## Abstract

**Abstract:**

The cardiovascular system is affected by a wide range of pathological processes, including neoplastic, inflammatory, ischemic, and congenital aetiology. Magnetic resonance imaging (MRI) and positron emission tomography (PET) are state-of-the-art imaging modalities used in the evaluation of these cardiovascular disorders. MRI has good spatial and temporal resolutions, tissue characterization and multi-planar imaging/reconstruction capabilities, which makes it useful in the evaluation of cardiac morphology, ventricular and valvar function, disease characterization, and evaluation of myocardial viability. FDG-PET provides valuable information on the metabolic activity of the cardiovascular diseases, including ischemia, inflammation, and neoplasm. MRI and FDG-PET can provide complementary information on the evaluation of several cardiovascular disorders. For example, in cardiac masses, FDG-PET provides the metabolic information for indeterminate cardiac masses. MRI can be used for localizing and characterizing abnormal hypermetabolic foci identified incidentally on PET scan and also for local staging. A recent advance in imaging technology has been the development of integrated PET/MRI systems that utilize the advantages of PET and MRI in a single examination. The goal of this manuscript is to provide a comprehensive review on the incremental value of PET and MRI in the evaluation of cardiovascular diseases.

***Main Messages*:**

• *MRI has good spatial and temporal resolutions, tissue characterization, and multi-planar reconstruction*

• *FDG-PET provides valuable information on the metabolic activity of cardiovascular disorders*

• *PET and MRI provide complementary information on the evaluation of cardiovascular disorders*

**Electronic supplementary material:**

The online version of this article (doi:10.1007/s13244-016-0494-5) contains supplementary material, which is available to authorized users.

## Introduction

Cardiovascular disease (CVD), which includes ischemia, inflammation, neoplasia, and congenital disorders accounts for 30 % of all deaths and is a leading cause of morbidity and mortality [1]. Early and accurate diagnosis is essential for the optimal management of these disorders. Several imaging modalities are used in the evaluation of cardiovascular disorders, each with their inherent advantages and disadvantages. Echocardiography is a widely available and portable modality, and there is good clinical familiarity with its application to cardiovascular disease. However, echocardiography is strongly operator-dependent, in some cases limited by acoustic window, and has limited tissue characterization [2]. Cardiac catheterization is the gold standard for evaluation of coronary vascular abnormalities and for interventional procedures. Computed tomography (CT) is a noninvasive technique which offers a three dimensional data set, is relatively operator independent, and provides good spatial resolution. Functional information is typically not obtained, but can be obtained if retrospective electrocardiographic (ECG)-gated acquisitions are utilized, although they are associated with higher radiation doses. Single-photon emission computed tomography (SPECT) is widely used in the evaluation of myocardial ischemia. Magnetic resonance imaging (MRI) and positron emission tomography (PET) are the two other commonly used imaging modalities.

The combination of PET and MRI often provides incremental information in the evaluation of cardiovascular diseases, more than each of the individual modalities separately. Based on our literature review, there is no comprehensive review on the incremental role of PET and MRI in evaluation of cardiovascular disorders, and therefore the goal of this manuscript is to provide one.

### Magnetic resonance imaging

MRI has several advantages including good spatial resolution, good temporal resolution, wide field-of-view, and multi-planar imaging/reconstruction capabilities, all without using ionizing radiation. In addition, MRI has tissue characterization capabilities due to inherent soft-tissue contrast, which can be brought out using different tissue-weighted sequences and augmented by administration of gadolinium-based contrast agents. MRI provides exquisite morphological information, and in addition, ventricular and valvular function can be quantified. There are numerous available MRI sequences that can be selected for a tailored approach each clinical scenario. (Table 1). Limitations of MRI include the cost, availability, and long duration of scanning, as well as a relative paucity of skilled readers and technologists. MRI cannot be used in patients with contraindications including those with metallic devices and those with claustrophobia. MRI is also avoided in patients with severe renal dysfunction due to the risk of nephrogenic systemic fibrosis [3].

**Table 1:** MRI sequences and their utility

Sequence	Planes	Description
Scouts	Sagittal, coronal, axial	Localizer
Single-shot Fast spin echo	Axial	Provides overview of morphology
Balanced Steady State Free Precession (SSFP)- Single shot	Axial	Provides overview of morphology
Cine SSFP	2-chamber, 3-chamber, 4-chamber, short-axis stacks	LV and RV volumes, ejection fraction, wall thickness, and motion
Black blood spin-echo - T1-weighted, - T2-weighted, - Fat saturated	2-chamber, 3-chamber, 4-chamber, short-axis stacks	Tissue characterization
T2*-weighted Sequence Planes Description T1- mapping Short-axis stacks Tissue characterization (fibros is, amyloid, deposition, iron) T2- mapping Short-axis stacks Tissue characterizaton (edema)	Short axis	Iron quantification
Diffusion	Localized to mass	Tissue characterization
Dynamic first pass perfusion	Short-axis stacks	Ischemia/infarct/microvascular obstruction/masses
2 min post contrast T1-weighted IR GRE	Short-axis stacks	For myocarditis, microvascular obstruction and masses
Delayed enhancement sequences	2-chamber, 3-chamber, 4-chamber, short-axis stacks	Evaluation of scarring and fibrosis
MR angiography	Coronal/sagittal/axial	Vascular anatomy
3d-whole heart SSFP	Axial/targeted to coronary arteries	Coronary artery anatomy Vascular anatomy

### Positron emission tomography

PET is based on the beta decay of radioisotopes that result in the emission of positron, a positively charged beta particle, which travels for few millimetres after emission and then collides with an electron resulting in annihilation of both and subsequent formation of two high-energy (511 kev) gamma rays. FDG (^18^F-fluoro-deoxy-glucose) is one of the most common PET isotopes, used for evaluating metabolism. In the cardiovascular system, FDG is used in the evaluation of myocardial viability, inflammatory/infectious disorders, neoplasms, and atherosclerosis. FDG-PET is very helpful for staging malignancies, optimizing biopsy location, guiding radiation therapy, assessing tumour response to therapy, and detecting tumour recurrence [4]. 11C acetate and 11C palmitate provide insight into myocardial metabolism. Rubidium 82 chloride, 13-N ammonia, and 15O water are useful in the assessment of myocardial perfusion [5, 6]. While PET has limited spatial resolution and anatomical localization, this can be overcome by adding CT (PET/CT), which also helps in attenuation correction—however, it adds to the radiation dose. Cardiac PET protocols and patient preparation are summarized in Table 2 [5–8].

**Table 2:** Patient preparation and protocol for cardiac ^18^F-FDG PET, NH4, and Rb-82 scans

	^18^F-FDG PET	NH4	Rb-82	^18^F-FDG PET
Reason	Viability study	Perfusion study	Perfusion study	Tumour/inflammatory lesion assessment
Diet	Low carbohydrate diet	Low carbohydrate diet	Low carbohydrate diet	High-fat, protein-permitted low carbohydrate diet for 24 hours Fasting for 6–12 hours
Check blood glucose	If blood glucose < 110 mg/dL AND not diabetic, oral glucose loading			Unfractionated heparin loading
	If blood glucose 130–200 mg/dL OR diabetic, administer IV insulin depending on blood glucose level			
	If blood glucose more than 200 mg/dL, notify physician (some institutes reschedule imaging)	If blood glucose more than 200 mg/dL, notify physician (some institutes reschedule imaging)		
Radiotracer administration	Administer 5–15 mCi of F-18 FDG when blood glucose below 150 mg/dL (preferable)	Administer 20 mCi NH4	Administer 40 mCi Rb-82	Administer 5–15 mCi of F-18 FDG
^18^F-FDG during and after administration	Patient should remain seated or recumbent to avoid muscular uptake			Patient should remain seated or recumbent to avoid muscular uptake
Start imaging	Wait 60 minutes, then scan for 10 minutes	Wait 10 minutes and scan for 5 minutes	Wait 10 minutes and scan for 6:30 minutes	Wait 60 minutes, then scan for 10 minutes

^18^F-FDG PET, ^18^F-fluoro-deoxy-glucose-positron emission; IV, intravenous; mCi, millicurie; Rb, rubidium; NH4, ammonium

### Cardiac PET and MRI

MRI and FDG-PET can provide complementary information on the evaluation of several cardiovascular disorders. The exquisite morphological information and tissue characterization capabilities of MRI can be complementary to the metabolic information of FDG-PET. The information obtained from PET and MRI acquisitions can be interpreted separately or can be fused using dedicated software algorithms. A significant challenge in fusion is achieving both spatial and temporal alignment, particularly in pulmonary and cardiac systems [9]. Cardiac motion can be improved by ECG-gating of both PET and MRI, while respiratory motion requires navigator gating for MRI or list mode for PET. Another challenge is the difference in acquisition times, which is 5–6 minutes for PET and just a few seconds for each MRI sequence. Studies have shown that fusion of PET and MRI can show misalignment as much as 2.0 ± 1.6 mm [10].

### Hybrid PET/MRI

Dedicated hybrid PET/MR systems have been recently introduced, which can potentially provide one-stop evaluation of cardiovascular disorders. The three commercially available PET/MR systems are (1) the Biograph mMR (Siemens Healthcare, Erlangen, Germany) in which PET detectors are integrated into the MR system with a single gantry using an avalanche photodiode-based technology, which allows simultaneous acquisition of PET and MR data; (2) the Philips Ingenuity TF PET/MR (Philips Healthcare, Andover, MA), which has a sequential design with two gantries positioned at each end of a single patient; and (3) the Discovery PET/CT 690 + Discovery MR 750 (GE Healthcare, Waukesha, WI), in which the PET/CT and MRI scanners are located in two adjacent rooms, and the patient is transferred between these two rooms using a detachable table (11). The advantage of these hybrid PET/MR systems is that they combine the strengths of MRI, i.e. morphological details, tissue characterization and spatial resolution, with that of FDG-PET, i.e., metabolic information. Compared to manual fusion, the integrated PET/MR systems provide improved spatial and temporal alignment. MRI image-based motion correction can also improve artefacts such as motion and partial volume effect in PET scanning. In addition, the hybrid technique results in significant time savings as compared with performing two separate examinations, improves throughput, and also reduces patient discomfort [9].

**Table 3:** Advantages of PET, MRI, and PET/MRI in different cardiac applications

Indication	PET	MRI	PET/MRI
Myocardial structure	Limited due to spatial resolution	Excellent due to good spatial resolution and several sequences	MRI contributes to improved spatial resolution
Ventricular and valvular function	Limited	Highly accurate ventricular function Valvular function can be quantified	MRI component provides the ventricular function
Metabolic activity of lesions	Excellent	No metabolic information	PET component provides information on metabolic activity
Artifacts - Motion - Respiratory - Cardiac - Partial volume	Artifacts can limit diagnosis	Several solutions available for artifacts	The MRI component can enable reducing artifacts
Cardiac masses	Metabolic activity	Morphological information Tissue characterization Functional quantification	Characterization of mass Localization of abnormal FDG activity in PET scan Assessment of cardiac involvement of mass seen in PET Local staging M staging Functional evaluation Response to therapy
Inflammatory disorders	Inflammatory metabolic activity	Morphological information Activity of lesion	Complementary information provided by different mechanism Morphological, functional, and metabolic information
Vasculitis	Metabolic activity	Disease activity Morphological information Vascular anatomy, complications	Evaluation of disease activity Comprehensive evaluation of vascular tree Lower radiation than PET/CT
Atherosclerosis	Plaque inflammation Targeted isotopes	Plaque component characterization Luminal evaluation	MRI localizes activity of PET Perfect spatial alignment Evaluation of lumen with MRI Lower radiation than PET/CT Targeted isotopes localized with MRI
Myocardial ischemia	Accurate Absolute quantification of MBF and CFT possible	Highly accurate Can quantify ventricular function Coronary anatomy can be evaluated	Comprehensive evaluation of perfusion along with vascular anatomy and function
Myocardial infarction	Accurate, reliable	Highly accurate Functional evaluation Complications Prognostic value	Complementary information

However, several challenges also exist in achieving widespread acceptance of this novel technology. This includes hardware issues such as photomultiplier tubes in the PET not working with strong magnetic fields, PET detectors producing magnetic field heterogeneities, and MRI surface coils causing unwanted attenuation interfering with gamma rays. Another important challenge is to develop a robust attenuation correction technique to accurately quantify the standardized uptake values (SUVs), since MRI signal does not contain information about tissue attenuation but reflects the distribution of protons. MR attenuation correction can be performed based on segmentation or atlas-based techniques. The segmentation approach is based on co-registration of the MR images to the PET transmission images using a surface matching technique, which is then segmented into several different classes. Using either a T1-weighted spoiled gradient echo, or T1-weighted 2-point mDixon, or ultra-short TE sequence, a three-segment model (air, soft tissue, lung) or a four-segment model (air, soft tissue, fat, and lung) can be used [11, 12]. In the atlas-based approach, an atlas is generated using a surplus of prior scans with corresponding known attenuation corrections. Each individual scan can be co-registered to the atlas by comparing each voxel to its nearest neighbours in the atlas. Then, the attenuation coefficients can be interpolated from the generated atlas [12].

### Applications of cardiac PET and MRI

In the following sections we will discuss the utility of PET and MRI in specific clinical cardiovascular scenarios. In each scenario, we will begin by discussing the current role of MRI and PET individually in the evaluation of these diseases and follow it up with the complementary information provided by both, as well as integrated PET/MRI technology (Table 3).

## Cardiac masses

Cardiac masses may be non-neoplastic (thrombus, hematoma, lipomatous hypertrophy, pericardial cyst), benign neoplastic (myxoma, fibroelastoma, lipoma, hemangioma, paraganglioma, rhabdomyoma, fibroma), or malignant neoplastic (metastasis, sarcoma, lymphoma, leukemia, mesothelioma). The most common cardiac mass is thrombus. Secondary tumours are significantly more common than primary tumours [13]. Myxoma is the most common primary tumour in the heart [14]. The role of imaging is (1) to characterize the mass, since it determines treatment strategy; (2) determine local staging, including extent of the tumour and involvement of adjacent structures, which is essential for surgical mapping; (3) to determine if chemo- or radiotherapy is required along with surgery; and (4) to assess response to therapy [15].

To characterize cardiac masses, MRI is performed using multiple tissue weightings (T1-w, T2-w, fat saturated sequences), diffusion, and various stages of contrast enhancement (early, dynamic perfusion, delayed enhancement). MRI features that suggest a benign or malignant lesion include margin, size, location, calcification, and pericardial effusion. Based on these factors, MRI has shown to be accurate in the prediction of lesion type (area under curve for two observers, 0.88 and 0.92, with p values < 0.0003 for agreement between the observers) [15]. Using long inversion times (>500 milliseconds), a thrombus can be distinguished from a neoplasm, since only a thrombus stays dark at this sequence. In addition, MRI also provides information on the extent of the mass, including involvement of adjacent structures. It also provides functional information, such as ventricular and valvular function, particularly if there is a valvular extension.

FDG-PET is also valuable in the evaluation of cardiac neoplasm. For evaluating cardiac mass, normal myocardial uptake is suppressed by fasting for at least 6 hours. Since malignant cells accumulate more glucose than normal cells do as a result of predominant glycolytic catabolism, significant uptake of F-18 FDG is indicative of malignant tissue. PET-CT has been shown to have 100 % sensitivity and 86 % specificity in differentiating benign from malignant cardiac tumours at SUV cut-off of 3.5 and 94 % sensitivity with 100 % specificity with SUV cut-off of 4.5. Benign tumours show only slight FDG uptake (2.8 + 0.9 vs 9.5 + 4.0 in malignant lesions). Extra-cardiac tumour manifestations may also be elucidated by whole-body PET/CT [16]. However, the CT component of PET is often inadequate to provide morphological information, which is essential for pre-surgical evaluation.

There are several scenarios in which the combination of MRI and PET provides additive information. This includes (1) the characterization of mass; (2) localization of abnormal FDG activity seen in PET scan; (3) assessment of cardiac involvement of mass seen in PET scan; (4) local staging, morphological information, and aggressiveness; (5) M staging; (6) functional evaluation; and (7) response to therapy, distinguishing scar tissue from recurrence.

### Characterization

**Fig. 1:**
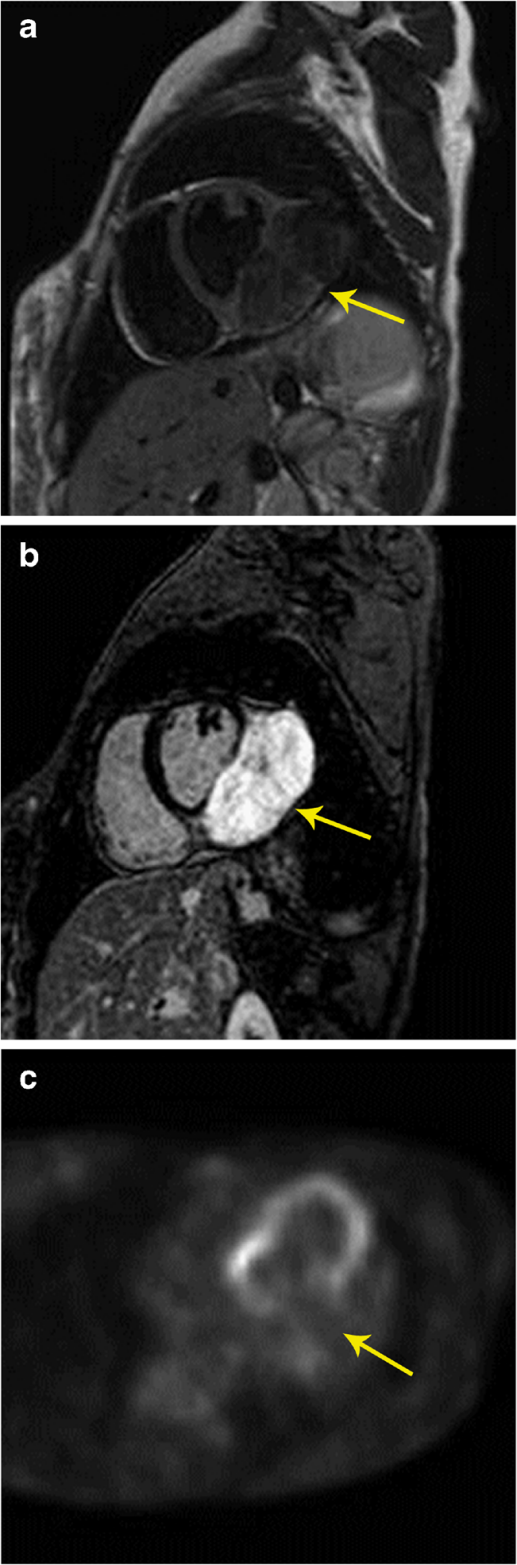
Characterization of mass. **a** 20-year-old female with irregular heart rate noted on physical exam, which eventually led to a cardiac MRI. Short-axis T2-weighted image shows intermediate to low signal 9 × 4 cm mass originating from the inferolateral basal to mid left ventricle (arrow). **b** There was no immediate contrast enhancement (not shown here), but there is intense delayed contrast enhancement in short-axis inversion recovery sequence (arrow). **c** Four-chamber FDG-PET scan shows no abnormal uptake in the mass, indicating a benign mass. The imaging findings were thought to be suggestive of fibroma. Based upon these findings, and the patient’s lack of symptoms, a choice was made to not intervene, and instead to obtain clinical follow-up as well as serial imaging. The patient has done well over six years, and the mass has shown no interval change in size

**Fig. 2:**
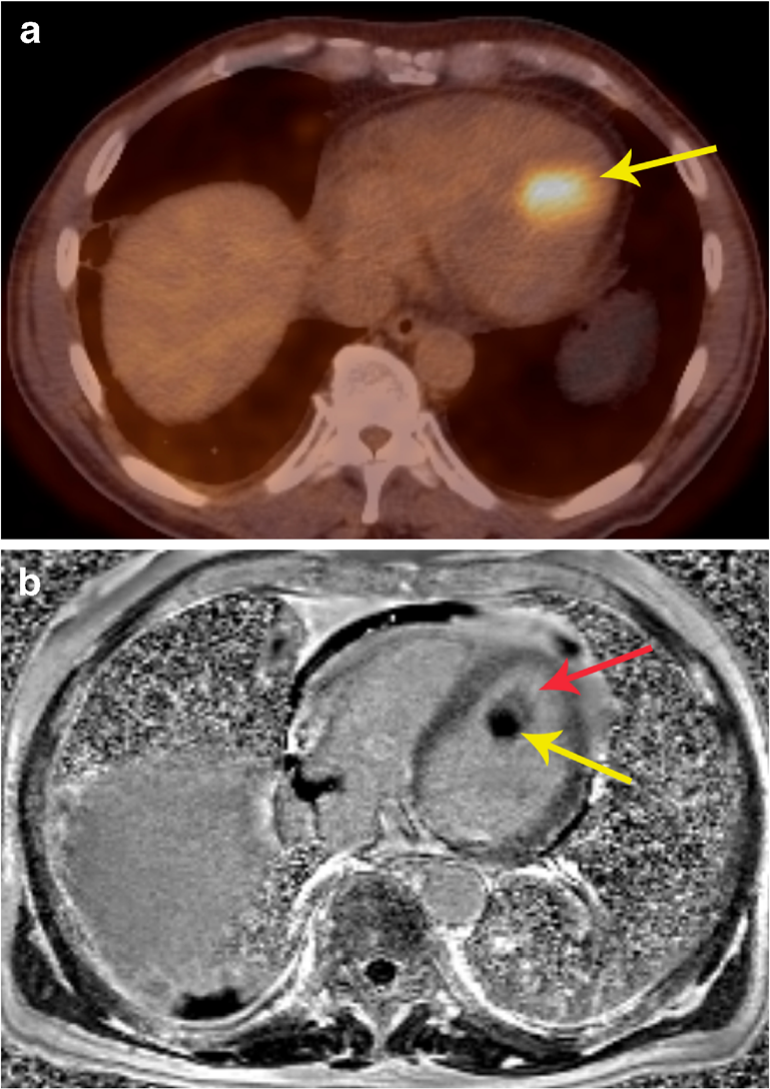
Characterization of mass. **a** Axial FDG-PET/CT scan in a patient with known thyroid cancer showed intensely hypermetabolic lesion (arrow) in the heart, which could not be localized clearly. **b** Four-chamber delayed enhancement cardiac MRI shows the mass to be entirely located within the left ventricular cavity. The mass has two distinct components, an enhancing component (red arrow) and a non-enhancing component (yellow arrow), which correspond to metastatic lesion and superimposed and bland thrombus, respectively. The patient was placed on anticoagulants in addition to chemotherapy

**Fig. 3:**
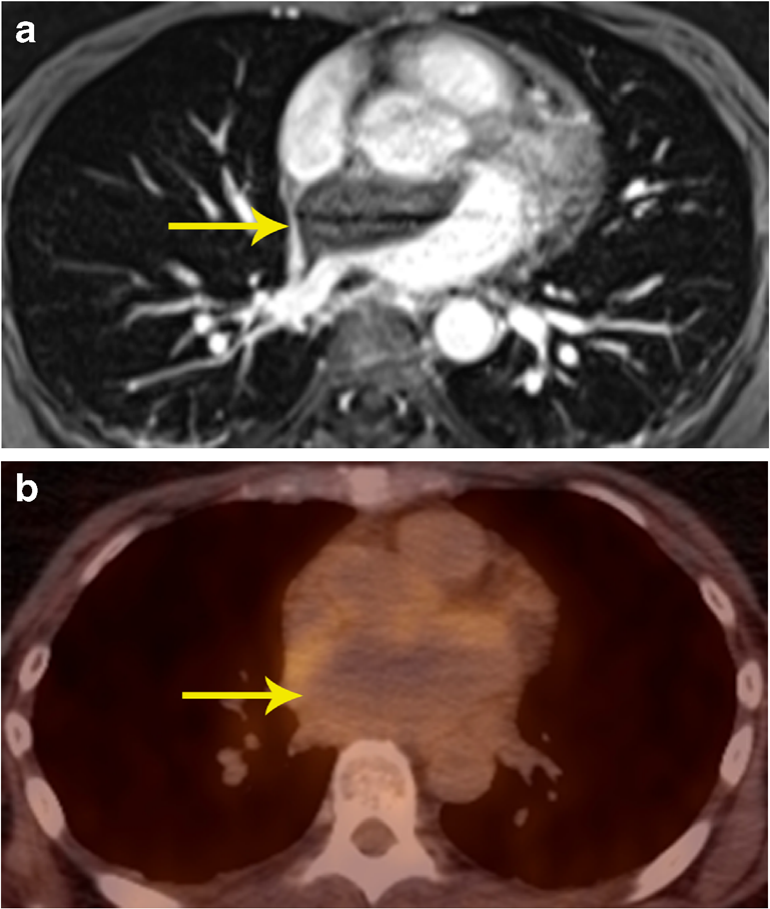
Characterization of mass. **a** Contrast-enhanced 3d SSFP MR image in a patient who presented with acute onset chest and back pain, shows a non-enhancing mass located in the atrial septum and left atrium extending to the mediastinum. **b** FDG-PET shows that there is no significant tracer accumulation in the mass. Based on the imaging findings, a neoplasm was excluded and a diagnosis of atrial hematoma was made. Follow-up imaging (not shown here) showed improvement

**Fig. 4:**
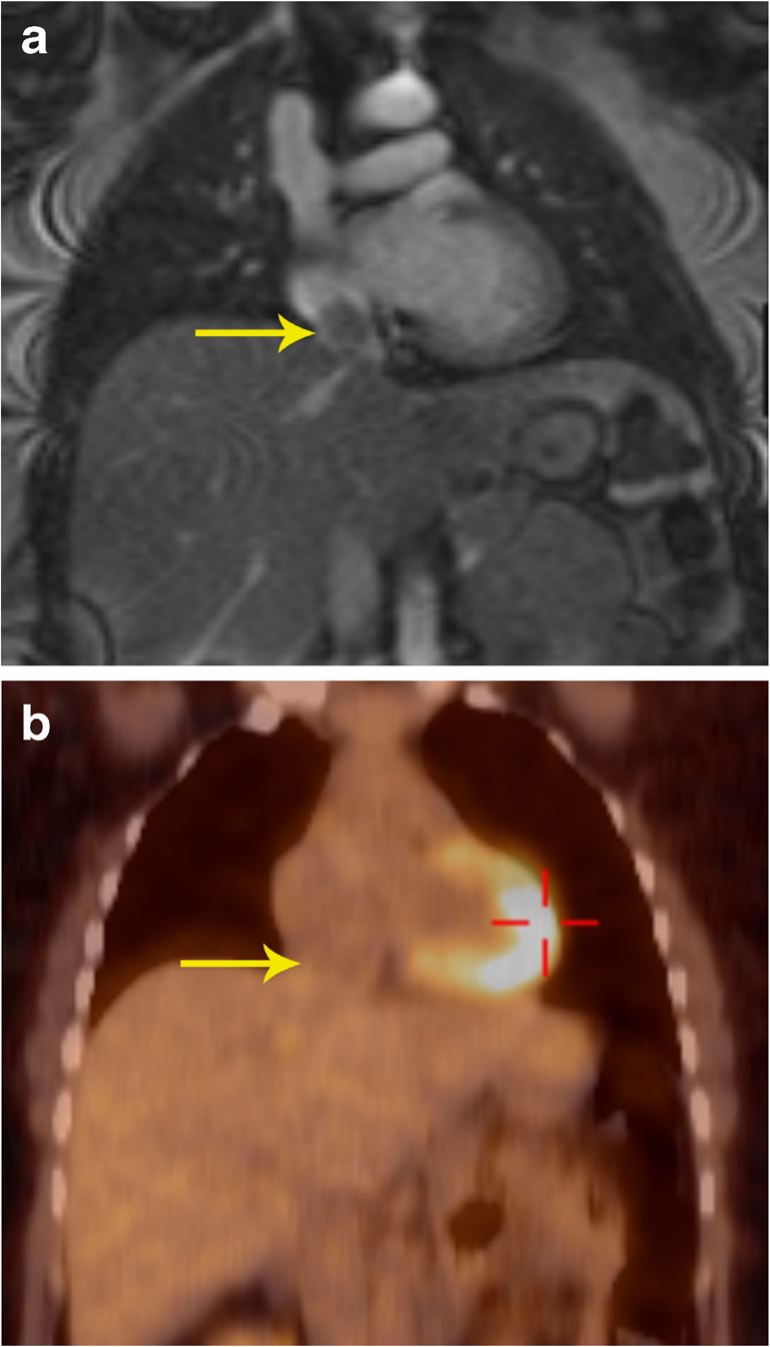
Characterization of mass. **a** Coronal first-pass perfusion MR image in a patient who presented with chest pain and IVC mass on echocardiography shows a mass that demonstrates contrast enhancement similar to the liver parenchyma. The mass also showed similar signal to liver in all other MRI sequences (not shown here). **b** Coronal FDG-PET/CT image shows no uptake in the lesion, indicating it is a benign process. This was proven to be a rare case of aberrant liver, which extended into the IVC

Although MRI and FDG-PET by themselves can characterize many tumours, the combination of both improves the diagnostic confidence in distinguishing malignant from benign lesions. If there is no FDG activity in a lesion, a malignancy can be excluded in most cases—an important piece of information that helps in management. False-positive FDG-uptake can be seen in inadequate patient preparation, inflammatory conditions (e.g. sarcoidosis), infection, abscess, surgical changes, radiation changes, and brown fat. Rarely, uptake can be seen in myxoma, which is also a false-positive finding [17] (Figs. 1, 2, 3 and 4). False-negative findings are seen in small lesions and carcinoid. In addition, specific isotopes such as 18 F-FDOPA, 18 F-FDA (fluorodopamine), 11C-hyroxyephedrine (11C-HED), and 68 Ga labelled may be useful in the evaluation of paragangliomas.

### Localization of abnormal activity seen in PET scan

**Fig. 5:**
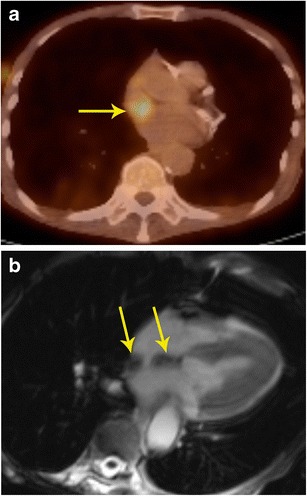
Localization of abnormal activity. **a** Axial FDG-PET/CT image shows a focus of high uptake adjacent to the SVC, which extended inferiorly. **b** Four-chamber SSFP MR image through the heart shows lipomatous hypertrophy of the interatrial septum (arrows), with sparing of fossa ovalis. This corresponds to the area of hypermetabolism in PET/CT. Although a benign lesion, this can occasionally show uptake in FDG-PET

**Fig. 6:**
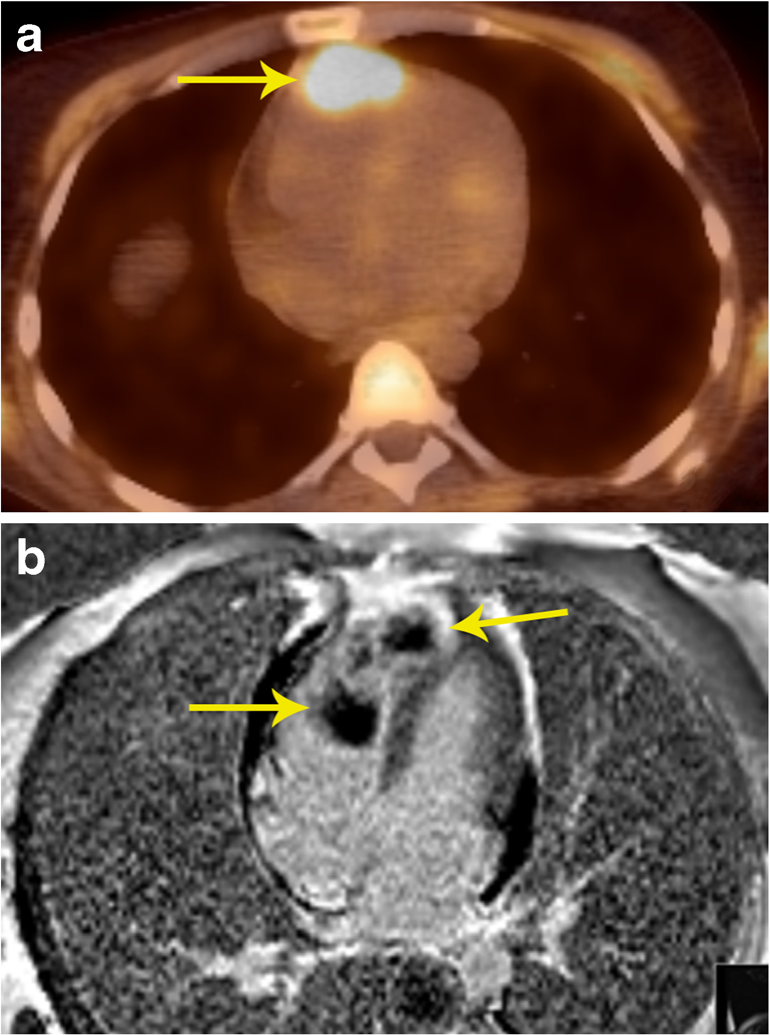
Localization of abnormal activity. **a** FDG-PET/CT image in a patient with breast cancer shows intense uptake in the anterior mediastinum abutting the heart. The exact location and extent of this lesion is not evident. **b** Axial delayed enhancement image shows that there is a heterogeneously enhancing mass in the right ventricle (arrow) that is extending to the right ventricular apex and also invading the right ventricular free wall

PET scans performed for evaluation of tumours or other purposes occasionally show abnormal focus of hypermetabolism in the chest, either in or adjacent to the heart. It is often not possible to accurately localize this lesion using PET or the corresponding low-dose CT scan. In such situations, MRI can provide additional information in localizing and characterizing these masses [3, 15] (Figs. 5 and 6).

### Assessment of cardiac involvement of mass seen in PET scan

**Fig. 7:**
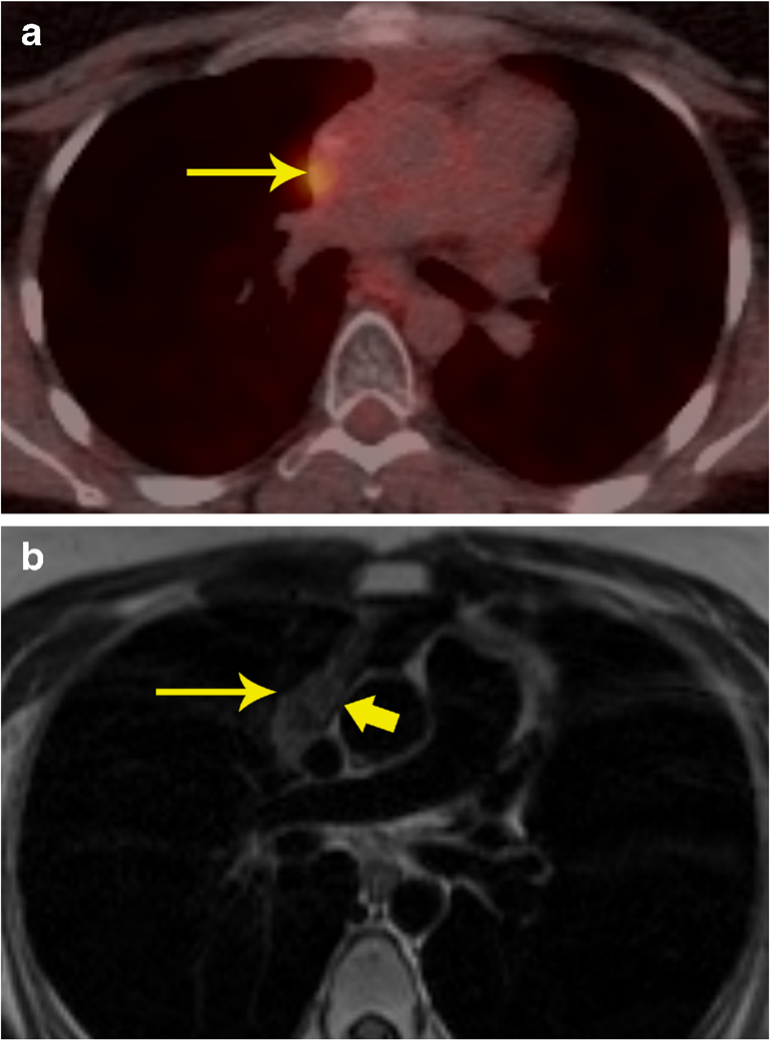
Evaluation of cardiac infiltration. **a** FDG PET/CT in a patient with lymphoma shows a focus of hypermetabolism adjacent to the SVC. The exact extent of this is not evident in the PET or the concomitant low-dose CT scan. **b** MRI was performed for evaluating cardiac extension. T1-weighted axial image shows that the mass (thin arrow) has a clear fat plane (thick arrow) with the SVC and cardiac structures, indicating that it is not infiltrating the cardiac structures

Further to the above utility, occasionally there are masses identified in PET imaging, which are in or adjacent to the heart (Fig. 7). It is important to identify the extent of involvement of the heart, particularly the involvement of cardiac chambers, myocardium, or pericardium. Correlative MRI images will allow for greater precision in cardiac mass anatomic localization [3].

### Evaluation of aggressiveness of lesion

**Fig. 8:**
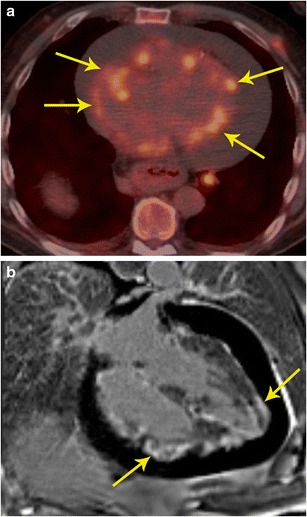
Local extension. **a** Axial FDG-PET/CT image in a patient with melanoma shows multiple hypermetabolic nodules on the cardiac surface (arrow). There is also pericardial effusion. **b** MRI was performed to better characterize the extent of these lesion, particularly to see whether there was myocardial involvement. Four-chamber delayed enhancement MRI shows the multiple pericardial masses extending to the myocardium. Diffuse heterogeneous myocardial enhancement (arrows) is seen, indicating diffuse infiltration

As discussed above, morphological information on the extent of tumour and involvement of adjacent structures is essential for surgical planning (Fig. 8). Neither PET nor the low-dose CT of PET/CT routinely provide this information and MRI is well suited for this purpose [3]. However, FDG-PET provides information on distal spread throughout the body [16].

### M staging

**Fig. 9:**
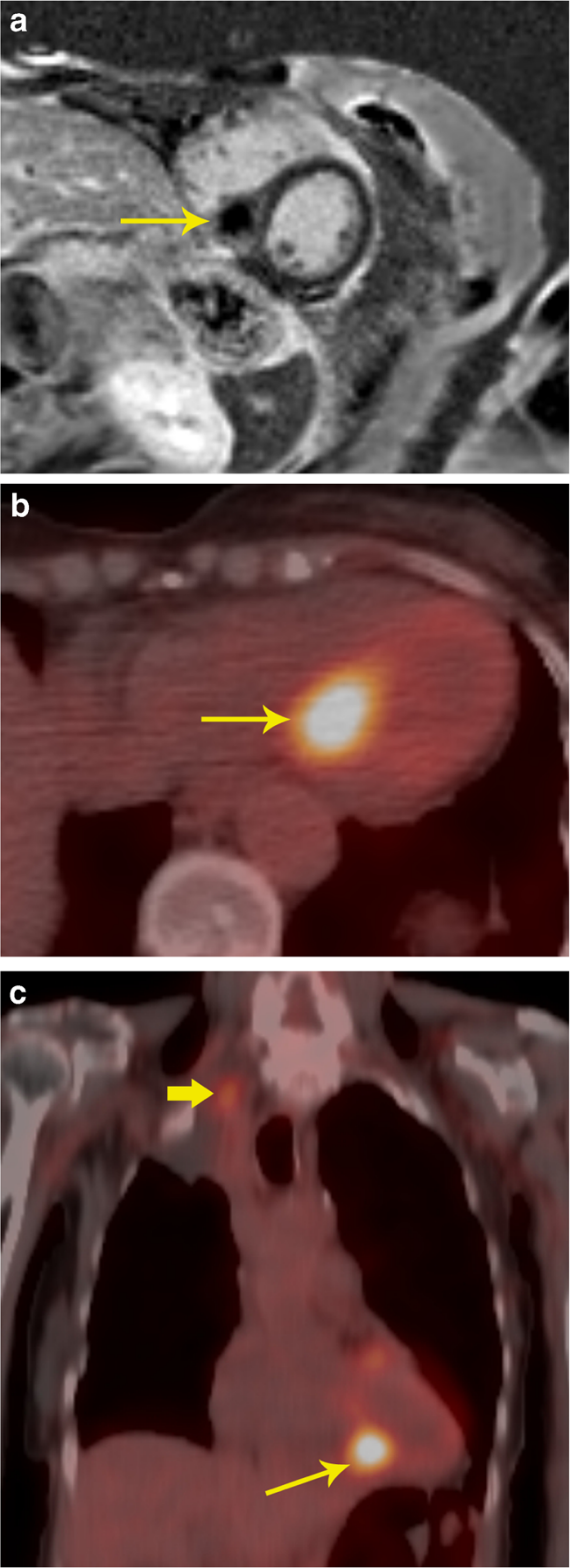
Staging. **a** Short-axis delayed enhancement MRI in a 72-year-old patient shows a mass in the ventricular septum (arrow) that has peripheral enhancement and central non-enhancement. **b** FDG-PET/CT image shows intense uptake in the mass in the ventricular septum, indicating that this is malignant. **c** Coronal FDG PET/CT image in the same patient shows the septal lesion (thin arrow). In addition, there was also a hypermetabolic right apical lung mass (thick arrow), consistent with lung cancer. The septal lesion is a metastasis. There were also metastatic lesions in the spine

**Fig. 10:**
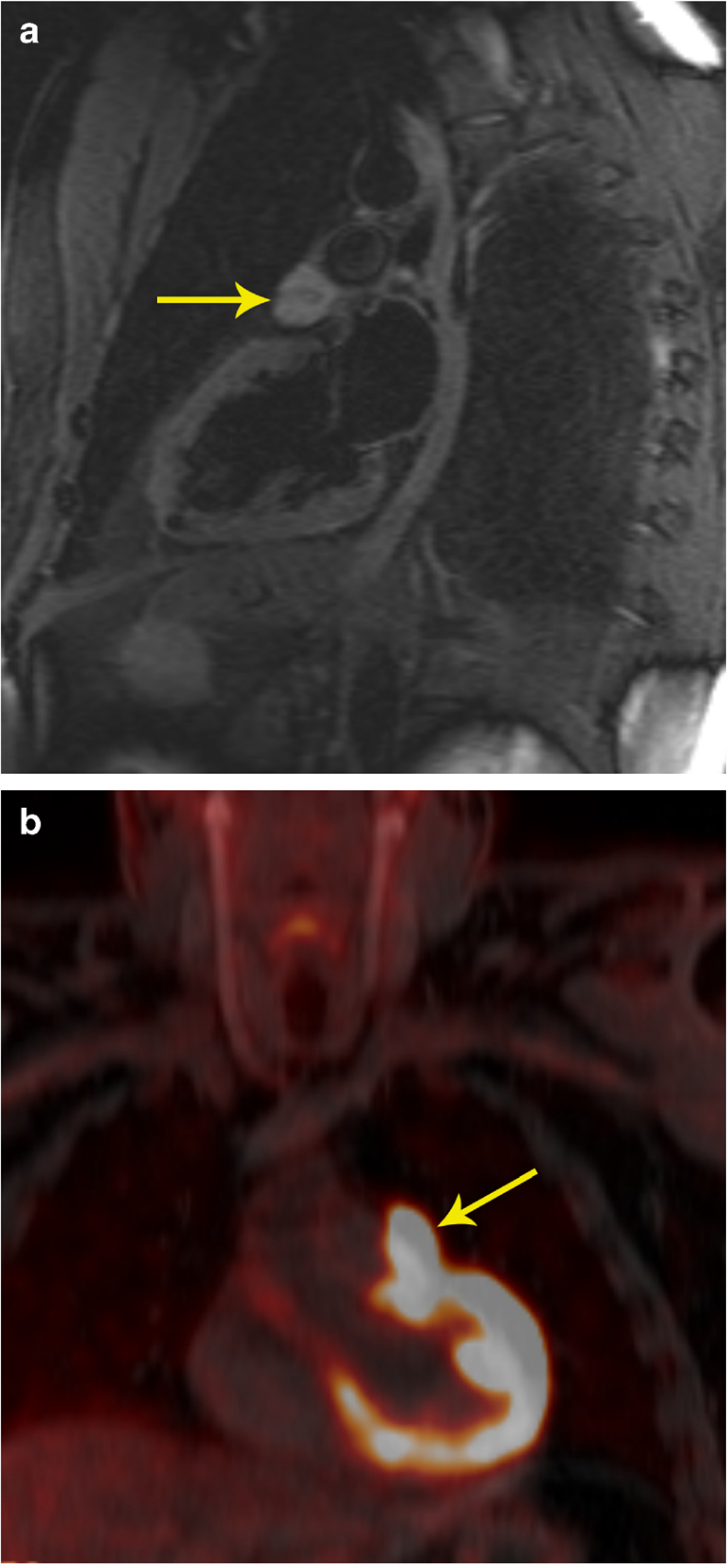
Characterization and staging. **a** 2-chamber STIR image obtained as a part of FDG-PET/MRI examination in a patient with cardiac mass shows a well-defined hyperintense mass adjacent to the left atrial appendage (arrow). This mass also showed intense contrast enhancement (not shown here). **b** The fused FDG- PET/MR image in the coronal plane shows intense uptake in the mass (arrow) adjacent to the left ventricle. This was shown to be a paraganglioma. The FDG-PETT scan also ruled out any other metastatic lesions, indicating that the lesion was benign.

Additional information obtained from PET can help in identifying non-cardiac primary or secondary neoplastic processes (Fig. 9). For example, paraganglioma is a tumour that is most often benign, but can be malignant in 10 % of cases. There are no specific imaging, or histological features to distinguish a benign or malignant paraganglioma. The only way to establish malignancy is to evaluate the presence of distal spread. Using FDG-PET, this can be determined and this helps in deciding the nature of the neoplasm (Fig. 10). Probst et al. reported the beneficial use of PET/MR in a patient with squamous cell carcinoma of lung with metastasis to the cardiovascular system [18]. Recently, whole-body MR diffusion-weighted imaging has been used in oncology. This technique has a high contrast between tumour and background tissues and is used for detection of tumour (primary, recurrence, secondaries), tumour grading, and therapy monitoring, particularly for bone metastases [19].

### Functional information

The MRI component also provides valuable functional information such as the involvement of crucial structures e.g., valve and papillary muscles. In addition, accurate quantification of ventricular and valvular function can be made.

### Response to therapy

Tumour response to therapy can also be evaluated using the metabolic activity of lesions with PET. PET and MRI are also useful in distinguishing scar tissue from recurrence [20].

A recent study showed that while FDG-PET and MRI separately had a sensitivity of 100 % and specificity of 92 % in determining cardiac malignancy, PET/MR achieved sensitivity and specificity of 100 % (Fig. 10) [21]. In summary, PET/MR provides valuable information on local and distant staging and also evaluates treatment response. With a hybrid PET/MR technique, this information can be obtained in a single study.

## Inflammatory disorders

The combination of PET and MRI is valuable in several inflammatory disorders, such as myocarditis, sarcoidosis, pericarditis, and valvar infections.

### Myocarditis

Myocarditis is characterized by an inflammatory reaction of the myocardium. The aetiology of myocarditis can be endogenous (toxins, autoimmune processes, etc.) or exogenous (bacteria, viruses, fungal, or parasites) [22]. MRI and PET provide complementary information for the assessment of myocardial inflammatory processes, including myocarditis [23]. MRI sequences used for myocarditis include the following: (1) T2-weighted (T2-W) images, which show myocardial oedema, an important hallmark of reversible inflammatory injury. Typically, myocardial oedema is seen locally, in a non-vascular distribution in subepicardial or mid-myocardial location. Occasionally, the oedema is diffuse and can only be detected by quantification of the T2 value, particularly when it is two standard deviations above that of skeletal muscle [24]; (2) early gadolinium enhancement, i.e. T1-weighted (T1-W) fast-spin echo images taken 1–2 mins after the injection of contrast agent, which shows enhancement in acute myocarditis due to hyperemia and capillary leak; and (3) delayed enhanced images taken 10–15 minutes after the injection of contrast agent, which shows enhancement due to fibrosis and necrosis [24–26], thus representing irreversible myocardial injury. The overall sensitivity, specific, accuracy, positive predictive value, and negative predictive value of MRI for myocarditis is 59 %, 86 %, 68 %, 89 %, and 53 %, respectively (24). On ^18^F-FDG PET scans, myocarditis presents as diffuse increased metabolic activity with areas of heterogeneity. Increased metabolic activity in myocarditis is secondary to microvascular and myocyte damage as well as changes in fatty acid metabolism [23]. Increased metabolic activity in myocardium is, however, nonspecific and can also be seen in congestive heart failure, right ventricular strain, and hypertrophy due to pulmonary hypertension [26].

**Fig. 11:**
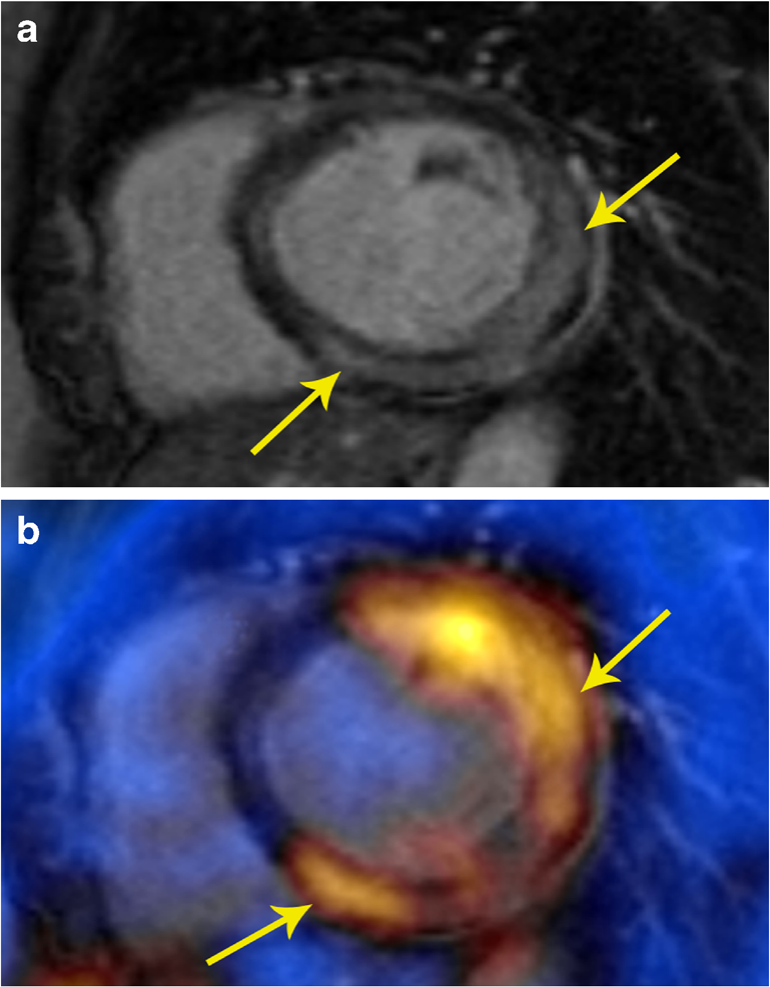
Myocarditis. **a** Short-axis delayed enhancement MRI in a patient with acute onset chest pain and elevated enzymes shows mid-myocardial enhancement in the mid lateral and inferior segments (arrow). **b** Fused PET/MR image obtained after glucose diet shows patchy areas of intense uptake in the lateral and inferior wall, indicative of active inflammation

The abnormalities in PET and MRI are based on different mechanisms, and hence they may provide complementary information in myocarditis. Other than functional information on the myocardial activity from MRI, PET can demonstrate activity of the inflammatory process in the myocardium (Fig. 11). MRI also provides functional information of the ventricles and valves. Since myocarditis usually affects younger patients in comparison to neoplastic cardiac masses, an argument could be made for the use of PET/MR as compared to PET/CT due to lower radiation exposure. PET and MRI can also be used for assessment of the response to medical treatment. However, other than short case reports, there is still lack of published reports on use of PET/MR in identification and follow-up of patients with myocarditis [27].

### Sarcoidosis

Sarcoidosis is a systemic granulomatous disease of unknown aetiology, likely due to an alteration in the immune response after exposure to an environmental, occupational, or infectious agent in genetically susceptible individuals [28]. Pathologic hallmark of sarcoidosis is non-caseating granuloma [29]. On MRI, acute phase of sarcoidosis manifests with myocardial thickening and oedema. Patchy myocardial oedema is seen due to active inflammation and is more commonly seen in the subepicardial region and less commonly in the mid-myocardial region. The oedema corresponds to areas of active inflammation, may present with ventricular arrhythmia or conduction disturbance, and usually responds to steroid therapy [30]. In the chronic phase, there may be wall thinning. Delayed enhancement is seen in up to 50 % of patients, in a mid-myocardial or subepicardial distribution due to replacement fibrosis [31]. Sarcoidosis often involves the septum (particularly basal portion) and left ventricular wall with rare involvement of the right ventricle and papillary muscles [32]. Wall motion abnormalities are also seen.

^18^F-FDG PET is also useful in the identification of cardiac sarcoidosis, with a sensitivity and specificity of 89 % and 78 %, respectively [29]. Recently, published data support the growing role of ^18^F-FDG PET-CT in the diagnosis, risk stratification, and assessment of response to treatment of patients with cardiac sarcoidosis [4].

**Fig. 12:**
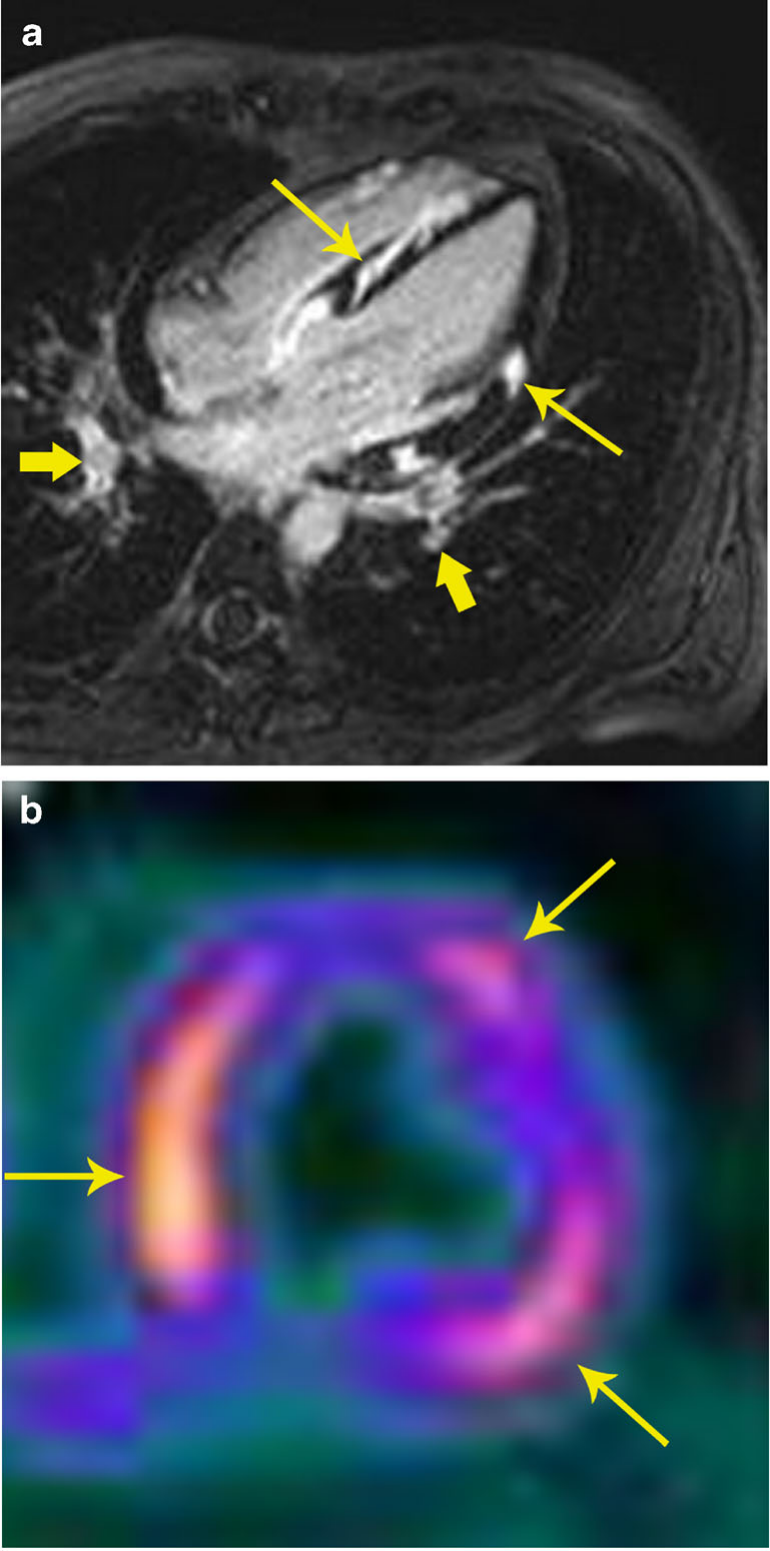
Sarcoidosis. **a** 32-year-old male admitted after a syncopal episode while shoveling snow. He was diagnosed with pulmonary sarcoidosis by transbronchial biopsy. Four-chamber delayed enhancement MRI shows patchy, diffuse areas of subepicardial as well as transmural areas of delayed post-contrast enhancement involving both ventricles (thin arrows). Bilateral hilar adenopathy was also seen (thick arrows). **b** FDG- PET after a fatty diet shows patchy areas of high uptake in the myocardium indicating areas of active inflammation

With PET/MR, the MR component evaluates for active inflammation, wall motion abnormalities, and fibrosis. FDG uptake indicates inflammation (Fig. 12). Since the disease detection is based on different mechanisms, the information obtained may be complementary, resulting in possible improved sensitivity in disease detection. PET/MRI also has the advantage of providing valuable information on the exact anatomical extension of sarcoidosis in the cardiovascular system, functional assessment of the myocardium as well as assessment of the disease activity and response to treatment (Fig. 12). This can help to improve the selection of available treatment options including medical management and cardiac resynchronization therapy [33].

**Fig. 13:**
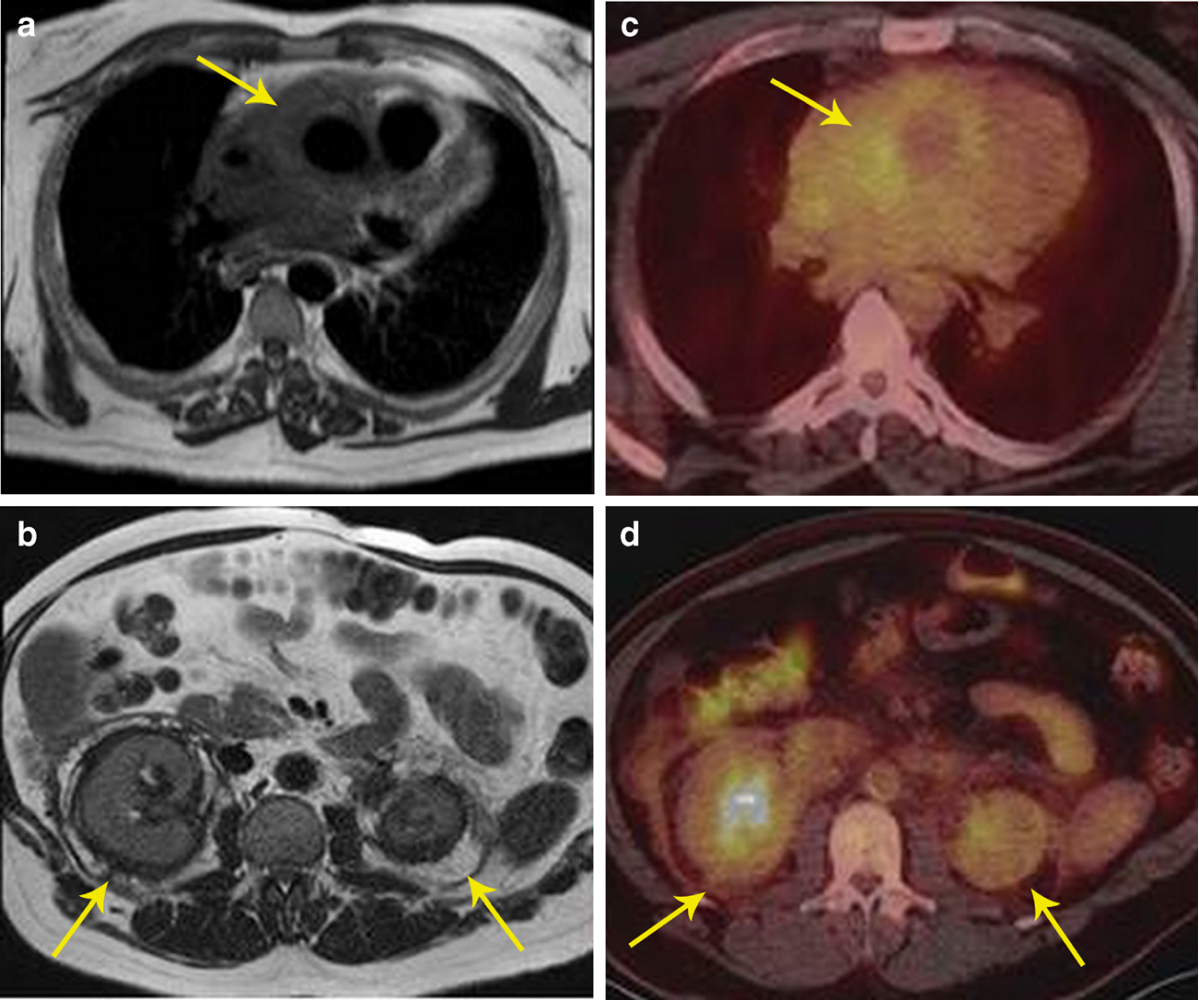
Inflammatory disease. **a** 46-year-old male with history notable for hypertension, bilateral orbital xanthogranulomas, and chronic low-grade fevers. As part of work-up, the patient had an aortic MRI that demonstrated diffuse confluent soft tissue within the mediastinum (arrow). **b** There were additional soft tissue changes as well as stranding surrounding both kidneys (arrows). **c** FDG PET/CT showed only minimal FDG avidity in the paraaortic soft tissue. **d** FDG-PET/CT also showed only minimally increased uptake around the kidneys. Bone scan (not shown here) was notable for diffusely increased uptake along long bones. Given these imaging and clinical findings, chronic inflammatory process with suggestion of mild activity at time of imaging was suspected; laboratory testing of orbital biopsy (CD68, CD163 positive, CD1a negative) led to a diagnosis of Erdheim-Chester disease

PET/MR is also useful in other inflammatory disorders, such as Erdheim Chester disease (Fig. 13), which is a rare disorder characterized by abnormal proliferation of non-Langerhans histiocytes, resulting in fibrosis that affects multiple organs.

### Pericarditis

**Fig. 14:**
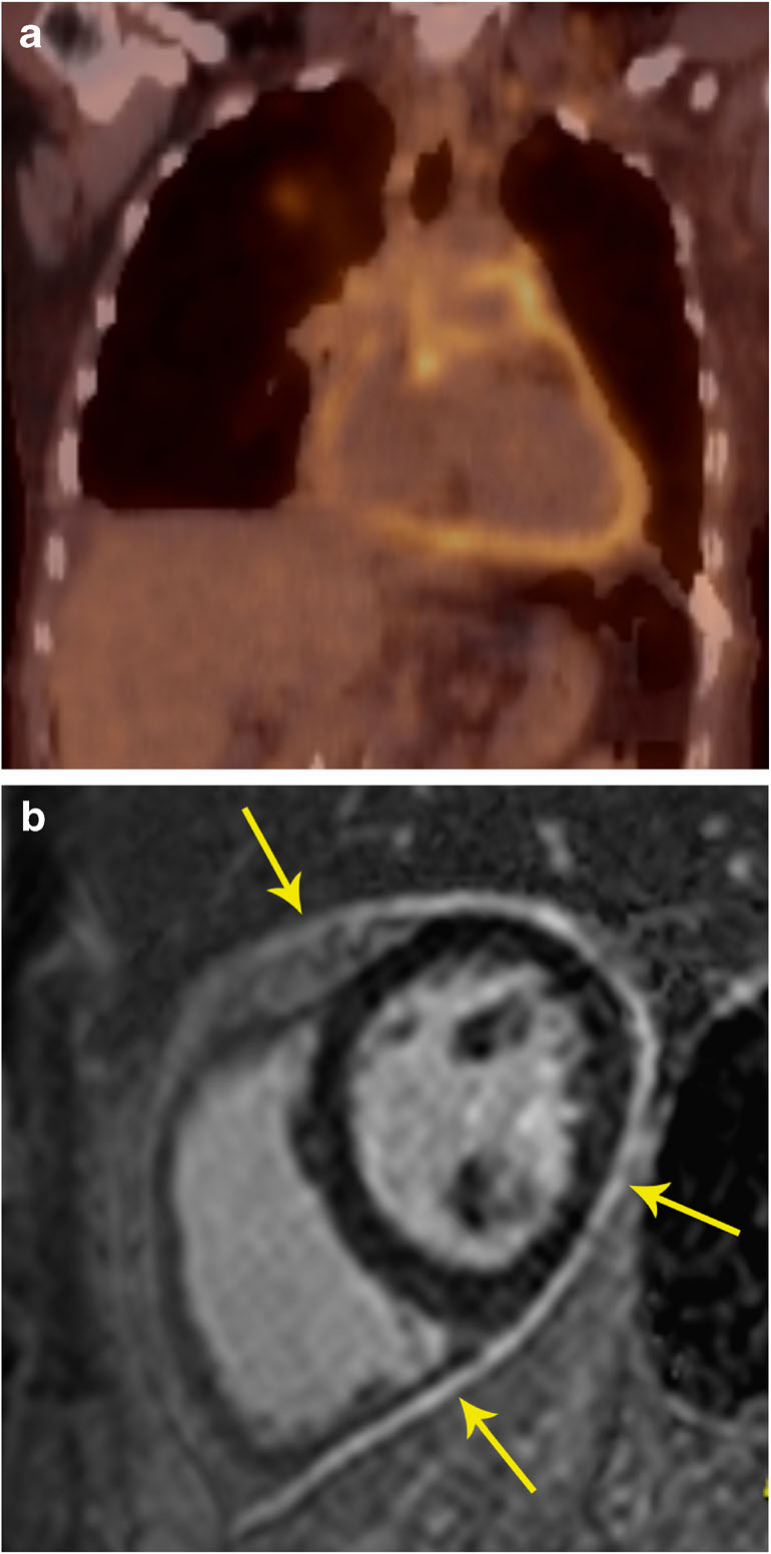
Pericarditis. **a** Coronal FDG PET/CT in a patient showed intense uptake in the pericardium. **b** Short-axis delayed enhancement MRI shows diffuse circumferential delayed pericardial enhancement (arrows), which is consistent with pericardial inflammation. There was also pericardial thickening in black blood images (not shown here)

Inflammation of the pericardium can have diverse aetiologies including idiopathic, autoimmune diseases, infections, post myocardial infarction, uremia, and radiation. Pericarditis can be of acute, chronic inflammatory, or chronic fibrosing types. MRI has become an important modality in the evaluation of pericardial disease. In acute pericarditis, there is pericardial thickening, pericardial effusion (Fig. 14a), and pericardial inflammation, which is manifested as delayed enhancement. In the chronic phase, pericardial thickening is present, but with lower amount of effusion and inflammation than acute type. In chronic fibrosing type, there is pericardial thickening with/without calcification and features of pericardial constriction may be seen. MRI is valuable in the evaluation of pericardial constriction, since it shows features of ventricular interdependence such as exaggerated septal flattening in real-time imaging (Movie 1), diastolic septal bounce, and abrupt cessation of diastolic filling. MRI is increasingly being used in the evaluation of pericardial inflammation, particularly in the context of transient pericardial constriction, which may be seen in the acute or subacute phase of pericarditis due to impaired pericardial distensibility. Although the standard treatment of pericardial constriction is aggressive pericardial stripping, in transient pericardial constriction, anti-inflammatory therapy (NSAIDs, colchicine, steroids) may be beneficial [34]. The presence of inflammation on MRI, even in the absence of clinical and serologic evidence of inflammation warrants continued therapy [34].

Inflammatory processes involving the pericardium demonstrate a mild-to-moderate FDG uptake (Fig. 14b), but occasionally no FDG uptake can be seen. In contrast, markedly increased FDG uptake is seen in proliferative neoplastic disorders that often corresponds with a localized mass [32]. A preliminary study showed that the FDG uptake is strong in tuberculous pericarditis compared to mild to moderate uptake in idiopathic pericarditis. In addition, hypermetabolic lymph nodes were also seen in tuberculous pericarditis [35]. Other causes of pericarditis can be distinguished based on history and FDG uptake patterns. Following surgery, there is diffuse and mild uptake and following radiation, there is diffuse uptake corresponding to the radiation port. Chemotherapy pericarditis is homogeneous and diffuse. Chronic pericarditis shows thickening and diffuse mild uptake with small or no effusion [35].

Although most cases of pericarditis do not require PET/MR, unique information provided by PET can be superimposed to MRI or CT and thus help to improve identification of inflammatory processes or masses in uncertain cases, to exclude other infectious foci, and aid in evaluation of response to therapy.

### Cardiac valve abscess

**Fig. 15:**
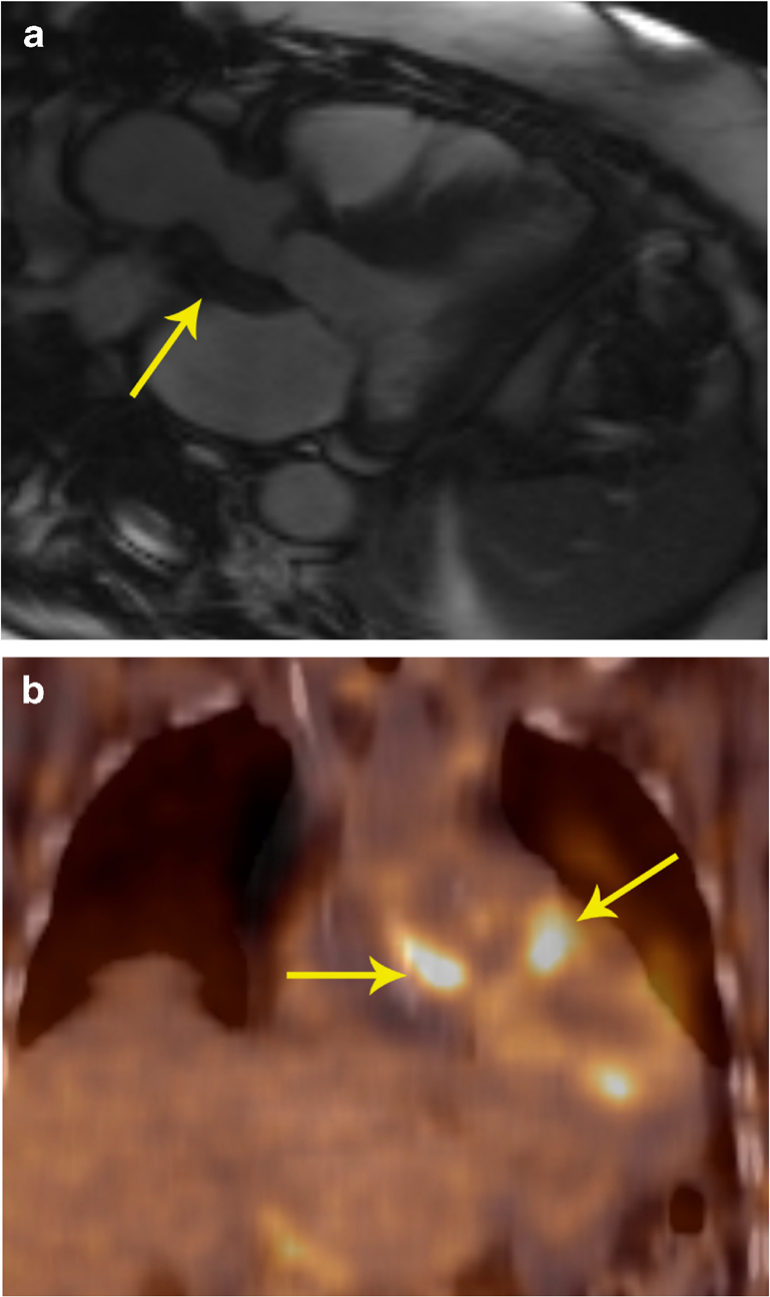
Perivalvular abscess. **a** Three-chamber Cine SSFP MRI image in a patient with history of bioprosthetic aortic valve replacement who presented with acute onset chest pain shows a soft-tissue intensity lesion surrounding the aortic valve and the aortic root (arrow). Contrast was not administered due to severe renal dysfunction. **b** Coronal FDG PET/CT in the same patient shows intense uptake along the lesion, which indicates that this is active inflammation/infection. Follow up MRI obtained two days later showed liquefaction and formation of abscess. This was treated surgically

Valve abscess is considered a complication of infective endocarditis (IE), either in a native or prosthetic valve, and requires urgent surgery with debridement of all infected and necrotic tissues. Echocardiography identifies only 40 % of surgically proven myocardial abscesses [35]. On MRI, morphological and functional evaluation of the valve can be performed along with quantification of valvular lesions such as regurgitation or stenosis. Inflammation or abscess is seen as soft tissue surrounding the valve. Inflammatory tissue has high signal on T2-weighted sequences (Fig. 15a) and abscesses show fluid signal with peripheral enhancement.

PET-CT has been shown to have higher sensitivity than echocardiography in the detection of valve abscess, showing intense uptake in the affected valves (Fig. 15b) [7]. Hybrid PET/MR imaging can aid in the characterization and localization of the high uptake zone on PET due to the excellent spatial resolution capabilities of MRI. The tissue characterization of MRI can also help to improve assessment of post-surgical cases in which high uptake can be expected due to post-surgical changes or residual/recurrent abscess. Information obtained from hybrid PET/MR can also achieve better differentiation of valve abscess from infective valve vegetations. Although there are some case series on the diagnostic value of PET-CT [36], there is no published report on the additional value of PET/MR in identification and management of cardiac valve abscesses [36]. MR or PET/MRI in patients with prosthetic valves can be challenging due to susceptibility artefacts. Sequences with lower susceptibility artefacts such as a conventional gradient-echo than a steady-state free precession (SSFP) should be used.

## Vascular disease

### Vasculitis

Vasculitis is inflammation of the vascular wall and can be classified based on the size of the affected vessels, location of vessels, and underlying cause. Aetiologies for vasculitis include autoimmune diseases, infection, and post chemotherapy [37]. Signs and symptoms are primarily dependent on the affected vessels and severity of disease. Although definitive diagnosis is established using biopsy, inflammatory markers and imaging are also helpful in diagnosis.

On MRI, active large vessel vasculitis presents with wall thickening, which may show oedema on STIR and enhancement with contrast. The oedema seen on STIR images, however does not show a consistent relationship with disease activity [38]. MR angiography provides comprehensive evaluation of the vasculature, able to identify abnormalities such as aneurysms and stenosis, and can be used to screen high-risk patients without any radiation exposure. MRI has been shown to have a sensitivity of 80 % and specificity of 90 % in the diagnosis of giant cell arteritis [39]. Since ^18^F-FDG accumulates in the macrophage-rich areas, vasculitis is seen in PET with high uptake in the vascular wall in a linear pattern. However, the tracer uptake for inflammatory disease is lower than for oncologic disease [40]. Also, PET/CT lacks the capability to show the vessel wall in the low-dose non-contrast CT. FDG-PET.CT has been shown to have an overall sensitivity and specificity of 80 % and 89 % for the diagnosis of giant cell arteritis when compared to reference clinical criteria [39].

**Fig. 16:**
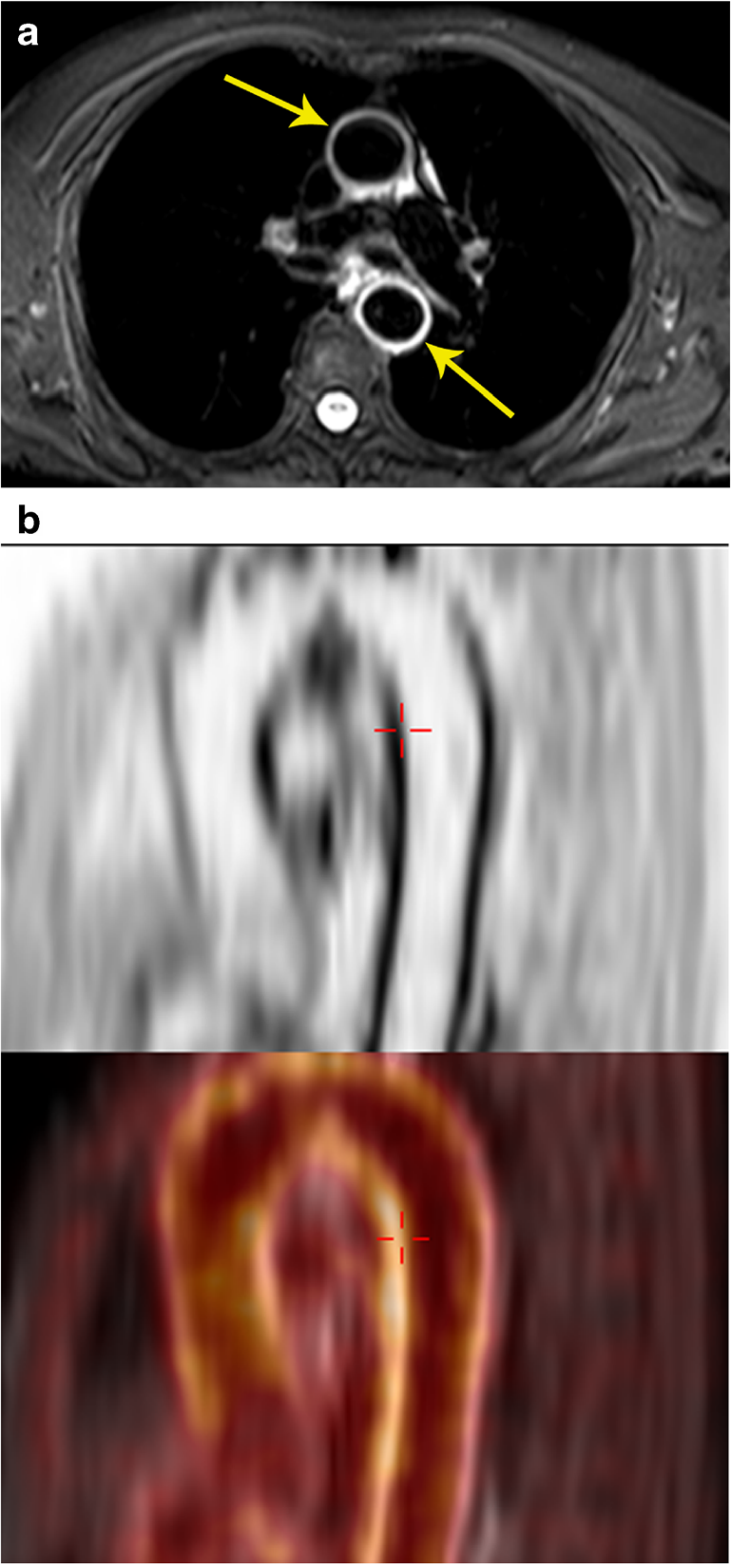
Vasculitis. **a** Axial STIR MRI image performed in a patient with suspected vasculitis following lung cancer chemotherapy shows an intense high signal in the wall of the ascending and descending aorta (arrows), which is indicative of active vasculitis. **b** Sagittal FDG PET image in the same patient shows the intense uptake along the wall of the thoracic aorta (arrow). **c** This high uptake is localized better to the aortic wall (arrow) using the fused PET/MR image (bottom)

PET/MR combines the anatomic information provided with MRI with the metabolic activity of PET, providing a reliable diagnosis of vasculitis with lower radiation dose than PET/CT, also at the same time enabling assessment of aneurysms and stenosis in the vasculature. In a pilot study on 14 consecutive examinations, PET/MR was found to be efficient in the evaluation of large vessel vasculitis and that only a combination of PET and MRI revealed the whole disease extent (Fig. 16). In MRI, abnormal segments were seen in 32/149, and in FDG 78/149, for an overall number of 83/149. Also, there was a strong and significant correlation between CRP and the number of vessel segments affected by inflammation in PET/MR (r = 0.86, p = 0.01) in contrast to PET or MRI only. Thus, PET and MRI are complementary for diagnosis and likely to be more accurate to assess the true extent of disease than either modality alone [41]. While the disease activity can be evaluated more accurately with PET, MRA provides a comprehensive evaluation of the vascular tree without the need for ionizing radiation [42].

### Atherosclerotic disease

Atherosclerosis is a diffuse, chronic inflammatory disorder characterized by deposition of lipid and fibrous products in the vessel wall, following complex biological processes. Current imaging techniques focuses on the detection of stenosis, grading of stenosis and evaluation of perfusion abnormalities distal to the stenosis. However, major events such as myocardial infarction (MI) and stroke are not produced by plaques with associated stenosis, but by non-stenotic plaques that get disrupted, the so-called vulnerable plaque. Hence, it is vital to be able to detect, quantify, and characterize the biological activity and stability of plaque at a subclinical stage. Identification of these high-risk features along with plaque burden will significantly help in the diagnosis and management of these patients [43].

High-resolution multi-contrast imaging of the vessel wall (T1-w, T2-w, PD, TOF) and dynamic contrast-enhanced angiography can characterize the specific tissue components of complex plaques, which helps to delineate biologic activity (Table 4) [43]. High-risk features of an atherosclerotic plaque include a large lipid-rich necrotic core, thin fibrous cap, endothelial denudation with superficial platelet aggregation, fissured/injured plaque and active inflammation. Stenosis > 90 %, superficial calcified nodule, intraplaque haemorrhage, outward positive remodelling and adventitial inflammation/neovascularity are the other features of vulnerability. Flow can be evaluated using time-of-flight sequence. Due to the smaller size, evaluation of coronary arteries is challenging, but this technique has been well established in the evaluation of carotid plaque [43].

**Table 4:** Characteristics of plaque components in high-resolution multi-contrast MRI

Component	T1-w	T2-w	PD	Contrast enhancement
Lipid	High	Intermediate	Intermediate	No
Fibrocellular	High	High	High	No
Calcium	Low	Low	Low	No
Necrotic core	Low	High	High	No
Fibrotic cap	Low	Low	Low	Yes
Haemorrhage	High	Low	Intermediate	No

FDG-PET has also been shown to be useful for assessment of inflammatory activity within plaque since macrophages in an inflamed plaque show avid FDG uptake and accumulation. High uptake has been shown both in symptomatic and asymptomatic plaques [44]. It has also been shown that there is a correlation between FDG uptake and extent of macrophage infiltration in a vulnerable carotid plaque [45]. However, low spatial resolution of clinical PET machines limits identification of small plaques and localization of high FDG uptake zones.

**Fig. 17:**
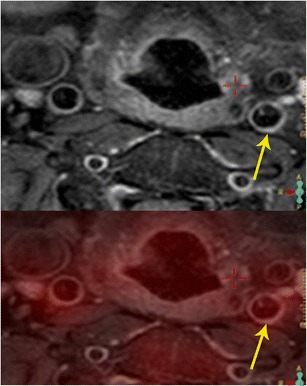
Carotid atherosclerosis. **a** Axial proton density-weighted MRI image (top) shows mild thickening of the wall of the left carotid artery (arrow) due to a plaque. **b** Axial fused PET/MR image at the same level shows uptake in the same area (arrow), indicative of plaque inflammation

With PET/MR, the high spatial resolution of MRI helps in localizing the metabolic activity obtained from PET (Fig. 17) [46]. Simultaneous acquisition also results in perfect spatial alignment. In addition, MRI also provides additional information on plaque components as described above [43]. Thus, the biological features of plaque obtained by PET provides additional information with multi-parametric plaque morphology assessment with MRI. The lumen can also be assessed at the same time for stenosis/occlusion. There is also potential for using targeted PET isotopes, which hone in on specific molecular targets of atherosclerosis, such as macrophage, or receptors of angiogenesis, vascular adhesion, and apoptosis. Some potential agents include 68 Ga-DOTATATE and 68 Ga- NOTA-RGD [46]. A recent study in HIV patients found congruence between PET/MR and PET/CT SUV values and improved delineation of outer and inner wall of carotid artery from MRI [47, 48]. MRI can be used for morphological follow-up and the combination can be used in drug trials.

## Coronary artery disease (CAD)

### Myocardial ischemia

Myocardial ischemia occurs when the oxygen requirement of part of myocardium exceeds the oxygen supply derived by coronary arteries, typically caused by coronary atherosclerotic disease. Identification of myocardial ischemia is essential in patients with coronary artery disease for management and for prognosis.

MRI is very useful in the evaluation of myocardial ischemia. Dynamic first-pass perfusion MRI performed at rest and stress has been shown to have high accuracy in the evaluation of myocardial ischemia. The CE-MARC trial showed sensitivity (86.5 %) and negative predictive value (90.5 %) of MRI to be statistically superior to that of SPECT (66.5 % and 79.1 %, respectively), while the specificity (83.4 % vs 82.6 %) and PPV (77.2 % vs 71.4 %) were non-statistically significantly superior [49]. Myocardial ischemia is seen as a hypointense perfusion defect at first pass perfusion imaging only at stress, with normal perfusion at rest.

Rubidium-82, Nitrogen-13 ammonia and 15 O labelled water are the PET isotopes used for perfusion imaging. PET has high accuracy, with sensitivity and specificity of 90 % in detection of obstructive coronary disease [50]. In addition, PET imaging is also valuable in quantification of myocardial blood flow (MBF) and coronary flow reserve (CFR), which is useful in multi-vessel disease, early changes of vasoreactivity and follow-up [9]. Also, PET findings have prognostic value [51].

With an integrated PET/MR system, simultaneous 13 N ammonia PET-MR myocardial perfusion has the advantage of increasing the accuracy of MR perfusion by the PET component, since the N-13 ammonia perfusion imaging has more coverage than stress perfusion MR. 13 N-ammonia PET has been shown to be an important cost-effective strategy in clinical decision making [52]. Utilizing MR attenuation correction data eliminates the radiation associated with CT attenuation data. Also, the examination time is shortened, since rest-phase 13 N-ammonia images are not required and are replaced by delayed enhancement images. In addition, MRI can also help address artefacts caused by motion or partial volume averaging. Ventricular function, global and regional, can be evaluated in the same study. A study which compared MRI with PET showed high accuracy of MRI in evaluating pathology with sensitivity of 91 % and specificity of 94 % compared to PET scan, but with significant underestimation of CFR [53]. Challenges of perfusion PET include lack of a good flow tracer for PET MPI, and short half-life of the isotopes, which necessitates having a cyclotron on site.

### Myocardial infarction

Myocardial infarction results from death of myocardial tissue due to blockage of coronary arteries. Imaging is useful in the diagnosis, prognosis, and evaluation of complications. An important contribution of imaging is detection of viable myocardium, which is dysfunctional but not dead, and hence can recover its function following revascularization.

Delayed enhancement imaging is highly sensitive and specific in the evaluation of myocardial infarct, both in acute (sensitivity of 99 %) and chronic phase (sensitivity 94 %) [53]. MI is manifest as a subendocardial or transmural pattern of enhancement confined to a vascular territorial distribution [54]. MRI is accurate in the quantification of myocardial function and scar, which has prognostic value. Dysfunctional segments with scarring > 50 % of myocardial thickness have been shown to have lower probability of functional recovery after revascularization [55]. MRI can also evaluate several complications of MI including thrombus, rupture, ventricular septal defect, pericarditis, etc. MRI has prognostic value and can also provide functional information [56]. The difference between oedema seen in T2-weighted images and the scar seen in delayed enhancement is considered the salvageable area by myocardial reperfusion [57].

**Fig. 18:**
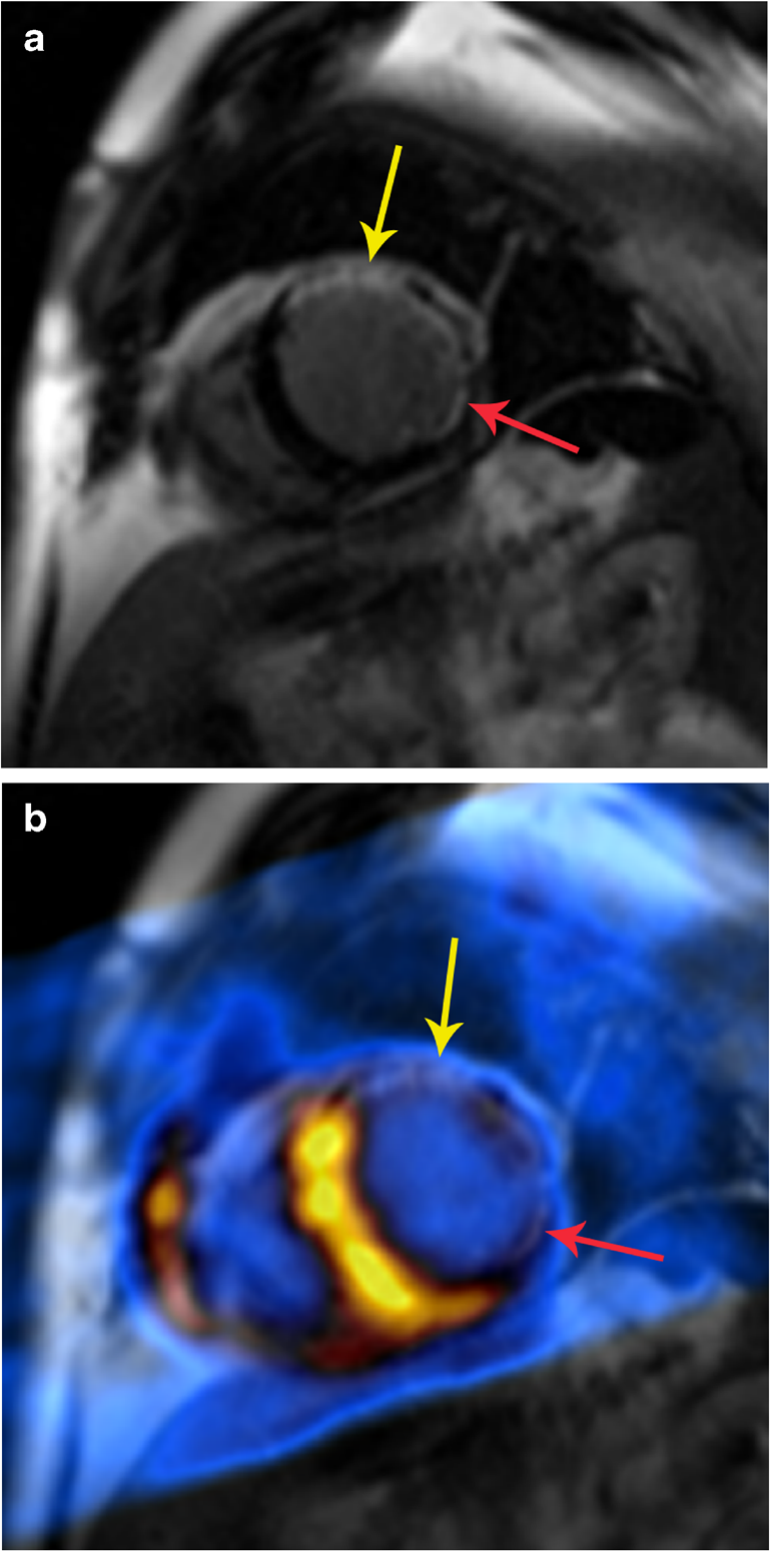
Myocardial infarction. **a** Short-axis delayed enhancement MRI in a patient with myocardial infarction shows a large transmural scar of the anterior wall (yellow arrow) and partial thickness scarring of the inferolateral segment (red arrow). Viability of this segment is borderline. **b** Fused PET/MR image shows no uptake in the anterior as well as inferolateral segments, indicating that these are not viable. Thus, the combination of these imaging modalities improves the diagnostic confidence

**Fig. 19:**
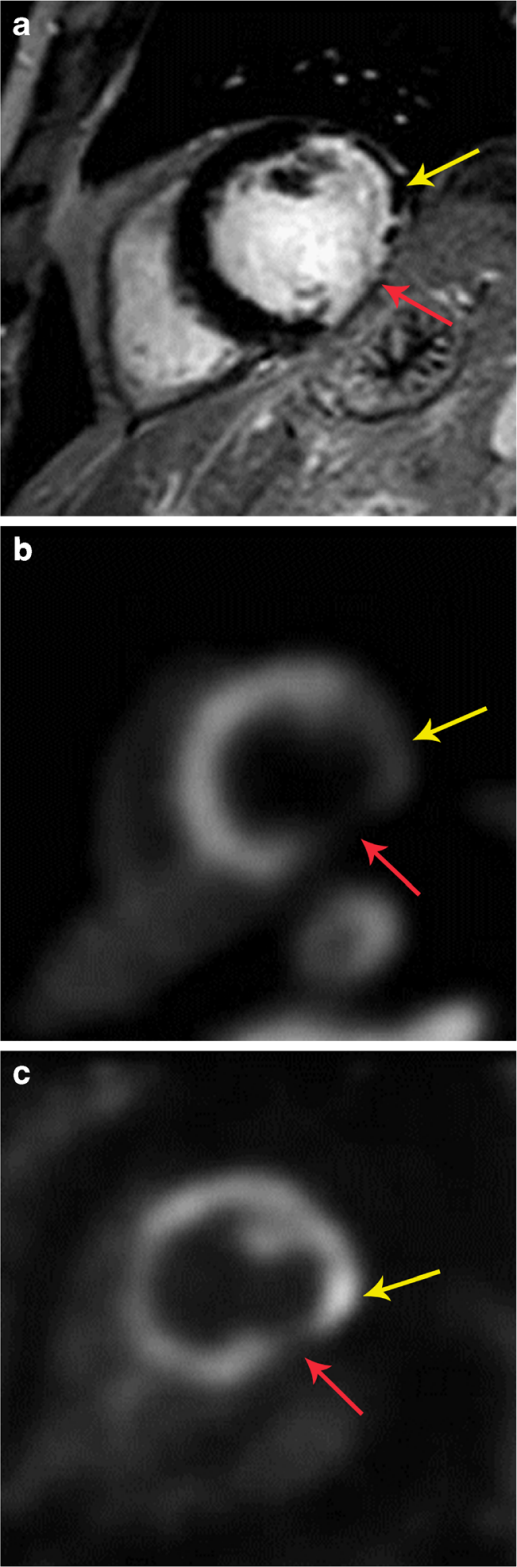
Hibernating myocardium. **a** 57-year-old male with coronary artery disease, status post multiple prior percutaneous interventions, with symptoms of angina at rest. Short-axis delayed enhancement MRI shows partial thickness scar in the anterolateral segment (yellow arrow) and near-full thickness scar in inferolateral segment (red arrow). **b** Rb-82 PET rest perfusion image shows perfusion defect in the anterolateral and inferolateral segments (yellow and red arrows, respectively). This was similar on stress images as well. **c** FDG-PET image shows no uptake in the inferolateral segment (red arrow) indicating non viability, while there is uptake in the anterolateral segment (yellow arrow), indicating hibernating myocardium

PET is also very reliable in the evaluation of myocardial viability, quantifying the degree of viability [58]. With PET, myocardial viability is detected by using a combination of perfusion imaging and FDG study [58]. Infarcted scarred tissue does not have ^18^F-FDG uptake (Fig. 18), while a viable hibernating myocardium has ^18^F-FDG uptake (Fig. 19). Hence, a matched defect, i.e. a defect in perfusion imaging and FDG scan, is indicative of a scar, while a mismatched defect, i.e. a defect in perfusion imaging, but normal FDG uptake, is indicative of hibernating myocardium [59].

A study using FDG and 13 NH3 PET and MRI showed that MRI can depict areas of non-transmural enhancement in viable areas of PET due to higher spatial resolution [60]. There was good correlation between the modalities for location and extent of infarct. Both modalities have comparable PPV for functional recovery after revascularization using 50 % thickness enhancement and 50 % FDG activity as cut off [58]. Both these techniques have high sensitivity but low specificity (63 %) in predicting functional recovery after revascularization, indicating that segments called viable do not always improve [61].

PET-MRI can also evaluate and characterize the heterogeneity of the scar tissue, since this is a marker for inducible monomorphic ventricular tachycardia [53]. The combination of PET SUV maps, metabolic function, delayed enhancement and T1 mapping may be better predictor of arrhythmia susceptibility than MR alone. The combination of PET and MRI can also assess other features such as decreased wall thickening, regional functionional abnormalities, lack of contractile response to low-dose dobutamine, preserved sub-epicardial myocardium, and preserved perfusion [9]. Both modalities provide prognostic information, although FDG-PET depends on mismatch between flow and glucose uptake, and MRI depends on infarct size.

### Ventricular function

Cardiac MRI is considered the gold standard in evaluation of myocardial function [60]. PET scan has significantly lower spatial and temporal resolution than MRI, limiting its accuracy in evaluation of cardiac function. Left ventricular function is calculated by algorithm-dependent assumptions on endocardial and epicardial border. Hence, the MRI component of PET/MR provides more accurate quantification of ventricular function.

### Coronary angiography

MRI also provides morphological information on the coronary arterial anatomy, including luminal stenosis [62]. Although inferior to CT, MRI can also be used for the evaluation of the anatomy of coronary arteries and evaluate coronary artery disease, with reasonably good accuracy. A recent multicenter trial showed sensitivity of 88 %, specificity of 72 %, PPV of 71 %, and NPV of 79 % compared to coronary angiography [62]. MR coronary angiography is performed using a 3d- SSFP sequence with navigator gating, images acquired during end-expiration and in diastole, with myocardial suppression by T2 prepared sequence. Thus, MRI provides concomitant anatomic information.

## Emerging and future applications

There are several emerging potential areas where hybrid PET/MRI is valuable in cardiovascular disorders. The most important application would be to utilize the high spatial resolution of MRI to localize the uptake of novel PET isotopes that are targeted towards specific pathophysiological process. This combines the anatomical detail of MRI with the highly sensitive metabolic information of PET. For example, 11C-labelled metahydroxyephedtrine (HED) and β-adrenoreceptor tracers can image defects of sympathetic innervation in cardiac disorders such as CAD, heart failure, and arrhythmias, thus helping in diagnosis, prognosis and selecting the appropriate therapy. 19 F- galacto RGE enables imaging of αvβ3 integrin, which is a marker of angiogenesis and also accumulates in the injured myocardium and thus can monitor effectiveness of therapies that aim to heal myocardium. Stem cell therapy can be directly visualized by reporter gene PET imaging [9].

## Conclusion

MRI and PET are clinically established imaging modalities, with complementary strengths which can be invaluable in the evaluation of a range of cardiovascular disorders. A combination of PET and MRI can provide synergistic information in several scenarios for the enhanced characterization of cardiovascular disorders. With use of hybrid PET/MRI scanners, this information can be obtained in a single scan.

## Electronic supplementary material

Below is the link to the electronic supplementary material.

Movie 1 Short axis real time cine imaging shows exaggerated septal flattening with inspiration indicating constrictive physiology in this case, likely inflammatory response. Thus MRI evaluated the morphology and also function. (WMV 5486 kb)
